# Comparison of the Main Constituents in Two Varieties of Proso Millet Using GC–MS

**DOI:** 10.3390/foods12122294

**Published:** 2023-06-07

**Authors:** Jana Pexová Kalinová, Jan Tříska, Karel Hořejší

**Affiliations:** 1 Faculty of Agriculture and Technology, University of South Bohemia in Ceske Budejovice, Studentska 1668, 370 05 Ceske Budejovice, Czech Republic; 2 Laboratory of Metabolomics and Isotopic Analyses, Global Change Research Institute, CAS, Belidla 986/4a, 603 00 Brno, Czech Republic; triska.j@czechglobe.cz; 3 Department of Chemistry, Faculty of Science, University of South Bohemia in Ceske Budejovice, Branisovska 1760, 370 05 Ceske Budejovice, Czech Republic; khorejsi@prf.jcu.cz

**Keywords:** amino acid, carboxylic acid, fatty acid, GC–MS, miliacin, proso millet, retinal, squalene, saccharide

## Abstract

Proso millet (*Panicum miliaceum*) is neglected in human nutrition. Thanks to the composition of the grains, millet is suitable for people with celiac disease and it is also useful in the prevention of cardiovascular diseases. For screening the substances in all plant parts of millet via GC–MS, two varieties, Hanacká Mana and Unicum, were used. Substances from the group saccharides, amino acids, fatty acids, carboxylic acids, phytosterols and others were identified in the roots, leaves, stems, and seeds. The highest level of saccharides was found in the stems (83%); amino acids in the roots (6.9%); fatty acids in the seeds (24.6%); carboxylic acids in the roots (3%), phytosterols in the seeds (10.51%); other substances, such as tetramethyl-2-hexadecenol (1.84%) and tocopherols (2.15%), in the leaves; retinal in the roots (1.30%) and squalene in the seeds (1.29%). Saccharides were the dominant group in all plant parts of proso millet followed by fatty acids. The dominant saccharides in all parts of the millet plant were sucrose, fructose and psicose. On the contrary, turanose, trehalose, glucose and cellobiose belonged to the least represented sugars. Additionally, amyrin, miliacin, campesterol, stigmasterol, β-sitosterol, and others were identified. Varietal variability can be assumed, e.g., in retinal, miliacin or amyrin content.

## 1. Introduction

Proso millet (*Panicum miliaceum* L.), also known as broomcorn millet or common millet, is a crop from the family Poaceae, which is probably one of the oldest crops in the world, originating in northern China [1]. Proso millet is ranked sixth among the world’s most important cereals [2]. The annual world production of millets accounts for about 30,463,642 t [3].

Grain of proso millet is rich in minerals (P 0.40 g/100 g, Ca 0.23 g/100 g, Zn 18.5 mg/kg, and Fe 25 mg/kg, [4]), dietary fiber, polyphenols, vitamins (niacin, B-complex vitamins, and folic acid) and amino acids (methionine, cysteine, leucine, and isoleucine), lecithin, and others. Of common cereals, proso millet has the highest lipid content (2.9%) in the grain after oats [5]. The lipids consist of 81% non-polar lipids, 14% glycolipids and 5% phospholipids [6]. Starch is the main carbohydrate in the grain of proso millet; it is suitable as a sizing agent in the textile industry [7]. Proso millet is gluten-free, has a low glycemic index and reduces the risk of type-2 diabetes, and cardiovascular disease [8]. Millet-based foods can include bread [9], cookies [10], muffins, couscous, snacks [11], or pasta [12].

Proso millet is a shallow-rooted, climate-smart, short-season crop (60–90 days) with high water-use efficiency, making it suitable for cultivation in both hot and dry environments worldwide and in a wide range of altitudes [13]. Therefore, proso millet is a promising rotational crop and can be grown as a second crop in a given year.

Proso millet possesses morpho-physiological traits, conferring greater adaptability and tolerance to abiotic stresses, especially drought and soil salinization [14]. Compared to the other cereals, proso millet’s nitrogen use efficiency (NUE) is 1.5–4 times greater than that of C3 cereals with a high leaf area index (LAI; 6.7), and a high radiation use efficiency (RUE; 2.5–4 g MJ^−1^) [15].

Proso millet can play an important role in global food security in the changing climate because water shortage is a significant threat to agriculture for the future [8]. The global population is predicted to increase to 9.3 billion in 2050, leading to serious food security issues [16]. The Food and Agriculture Organization of The United Nations (FAO) has identified proso millet as one of the future smart crops of the twenty-first century [17]. Millet cultivation could support agrobiodiversity and provide a regionally available source of highly nutritious cereal grain [18].

Nowadays, proso millet grain is primarily used for feeding birds and used as livestock feed in developed countries and in some developing countries, it is used as food. Proso millet is used to produce liquors and beers or fermented beverages in Africa and Asia. Green plants are excellent fodder for cattle and horses and are also used as hay [19]. However, information on the feed value of millet forage or silage is limited.

The straw residuals are often considered an agricultural by-product. Agricultural residues represent one of the most important energy-rich resources. Biomass contributes about twenty-five percent of the world’s energy requirement, which is equivalent to twenty million barrels of fuel oil per day [8]. There is still a lack of studies describing the composition of the various millet parts that would allow their broader use in industry. Our interest in millet plants is also based on studies aimed at the recovery of agricultural residues, including various morphological parts of plants, in which various lipophilic substances with valuable nutraceutical properties have been identified, such as phytosterols, steryl glucosides, tocopherols, etc. Therefore, the main task of our study is to compare the compounds contained in different proso millet varieties.

## 2. Materials and Methods

### 2.1. Material

Proso millets (*P. miliaceum* L., Hanácká Mana and Unicum varieties) were used for the experiment conducted on plots (a plot 10 m^2^) in Ceske Budejovice, Czech Republic (48°57′42″, 14°28′05″, 380 m elevation, annual mean air temperature 9.2 °C, annual sum of precipitation 582 mm, annual sunshine duration 1791 h, sandy-loam soil, and the content of main nutrients in the soil: phosphorus 168 mg/kg, magnesium 102 mg/kg potassium 162 mg/kg, calcium 1060 mg/kg, and pH 5.5). The seeds of both varieties of proso millet were obtained from the official dealer of proso millet in the Czech Republic, Oseva UNI a.s. The varieties were chosen because they are original, available, and often grown in the Czech Republic. Proso millet was sown on 28 June 2021 in 25 cm wide rows, after a cereal fore-crop, using a seed drill with 250 seed/m^2^ density for precise drilling. No chemical treatment was performed during the growing season. Before harvest, thirty whole plants at the stage of full ripeness (110 days old plants for for Hanacká Mana and 113 days old plants for Unicum) were sampled in three replicates. The plant samples were manually cleaned to remove all foreign material, divided into roots, leaves, stems and seeds, dried at 45 ± 1 °C, and ground to powder with a mortar and pestle.

### 2.2. GC–MS Analysis 

Ground samples of individual plant parts of both varieties of proso millet (1 g) were extracted with 15 mL of dichloromethane: methanol (2:1). Extraction was performed in an ultrasonic bath (Hwashin Technology Co., Yeongcheon, Republic of Korea) at 50 °C for 60 min at maximum power. From the settled mixture, 1000 µL of the clear extract was taken into a glass vial (2 mL), and after evaporation of the solvents (stream of nitrogen at 40 °C), the residue was dissolved in 75 µL of dry pyridine and then derivatized with 75 µL of N-methyl-N-(trimethylsilyl)trifluoroacetamide (MSTFA) for 1 h at 70 °C. After derivatization, 250 µL of hexane and 20 µL of Fluoranthene-D10 solution (25 mg/L) were added to the derivatization mixture as the internal standard (IS). The limits of detection (LOD) and quantification (LOQ) for IS are given in Table S1.

After extraction and derivatization of the sample, 1.0 µL of extract was injected into a Thermo Scientific Trace 1310 gas chromatograph (Thermo Scientific, Waltham, MA, USA) connected to a TSQ 8000 mass spectrometer (Thermo Scientific) equipped with triple quadrupole analyzer using a split/splitless injector and Al 1310 autosampler. Rxi 5Sil MS capillary column (Restek, Bellefonte, PA, USA) with an inner diameter of 0.25 mm, a length of 30 m and a film thickness of 0.25 µm was used to separate the analytes. The carrier gas flow (helium) was kept constant at 1 mL/min with the split ratio 1:25 after 2 min delay. The split/splitless injector temperature was maintained at 290 °C, and the column temperature was programmed as follows: see Table 1.

The MS transferline was set at 290 °C. The analytes leaving the capillary column were ionized via electron impact at 70 eV at an ion source temperature of 280 °C. Mass spectra were acquired in full scan (40–800 *m/z*) for qualitative and quantitative analysis. The measurement results were evaluated by comparing the areas of individual peaks (substances) with the area of the added internal standard and by determining the area (representation) of each peak to the total area of all peaks in the sample. Most compounds were identified by comparing the measured spectrum with the library spectrum (NIST 2011). The percent of an individual compound or a group of compounds in the sample was calculated according to following formula:

Percent of compound A (%) = Peak area of compound A/Total area of all peaks × 100

### 2.3. Chemicals and Reagents

Following chemicals and reagents were used: dichloromethane (for residual analysis, purity ≥ 99.9%, Chromservis, Prague, Czech Republic), methanol (HPLC ULTRA LC-MS grade, purity ≥ 99.9%, VWR International, Stříbrná Skalice, Czech Republic), n-hexane (for residual analysis, purity ≥ 95%, Fischer Chemicals, Hampton, NH, USA), pyridine (anhydrous, purity ≥ 99.8%, Sigma-Aldrich, Prague, Czech Republic), N-Methyl-N-Trimethylsilyl Trifluoroacetamide—MSTFA (Restek Columns, Bellefonte, PA, USA), Fluoranthene-D10 (purity ≥ 98%, Sigma-Aldrich), and N2 (purity ≥ 99.5%) produced by Genius 1022 nitrogen generator (Peak Scientific, Inchinnan, Great Britain).

### 2.4. Statistical Analysis

Data were subjected to analysis of variance (ANOVA) using Statistica 12 statistical software. The significant differences among groups were determined via the Tukey HSD test (*p* < 0.05).

## 3. Results

The chemical composition of the proso millet parts was studied using GC–MS analysis, and the identification of the main compounds is summarized in Table S2. The extracts analyzed via GC–MS were identified using the NIST 08 Library [20]. Match Factor in the range of 800–900 is a good match and in the range of 700–800 is a fair match according to the NIST 08 Library. The lower value of Match Factor can be caused by the imperfect shape of the peaks at lower concentrations of the substances and their incomplete separation. Value under the range of 700 does not provide reliable data for identification, which is the case for the following substances: some hydrocarbons, 3-hydroxy-oxypregnan-one, and tetracosanoic acid, bis[(TMS)oxy] propyl ester.

Before GC–MS analysis, we used a very specific method of extraction that is certainly very selective and may not be the best for each class of compounds. It is therefore important to remind here that the results are based on a partial view of the complete composition of proso millet plant. The determined amino acids are probably only the free ones since no protein hydrolysis was performed and the lipids were analyzed without saponification.

In general, the vegetative plant parts (roots, stems, and leaves) of proso millet were mainly composed of saccharides and fatty acids, followed by amino acids (Table 2). Seeds of proso millet also contained the most saccharides and fatty acids followed by phytosterols (Table 2). The largest proportion of saccharides was detected in stems, followed by roots, leaves and seeds. In the seeds, the sum of the peak areas of saccharides was 0.55 times of the mean for both varieties in the stems. The value of the sum of the areas of saccharides peaks in the roots was on average 0.86 times of the value in the stems and in the leaves was 0.64 times of the value in the stems.

The largest proportion of fatty acids was detected in seeds. The value of the sum of the areas of fatty acids peaks in the roots was on average 0.45 times of the value in the seeds, the value for stems was 0.34 times of the value for the seeds and the value for leaves was 0.60 times of the value for the seeds. However, the differences between parts were not statistically significant.

The largest proportion of phytosterols was also detected in seeds. The sum of the areas of phytosterols peaks in the roots was on average 0.26 times of the value for the seeds, the value for stems was 0.09 times and the value for the leaves was 0.41 times of the value for the seeds.

Roots showed the largest proportion of identified amino acids. The sum of the areas of amino acids peaks in the leaves was on average 0.26 times of the value for the roots, the value for stems was 0.09 times and the value for the leaves was 0.41 times of the value for the roots. However, the differences between parts were not statistically significant.

Roots also showed the largest proportion of identified carboxylic acids. The sum of the areas of carboxylic acid peaks in the stems was, on average, 0.64 times the value for the roots, the value for leaves was 0.45 times, and the value for the seeds was 0.45 times the value for the roots. However, the differences between the parts were not statistically significant.

There is a noticeable difference between the varieties in the proportion of identified saccharides in the stems; compared to the Unicum variety, the Hanácká Mana variety had a higher proportion of saccharides (Figure S1). The sum of the saccharide peak areas was 1.3 times larger in the Hanácká Mana variety than in the Unicum variety. On the contrary, a larger proportion of amino acids, fatty acids, phytosterols, carboxylic acids and miscellaneous was found in the Unicum variety. The Unicum variety had 4.3 times larger sum of amino acid peaks in the stems, 2.5 times in the leaves and 1.5 times in the seeds than the Hanácká Mana variety. Only in the roots, 3.3 times larger peak areas of amino acids were determined for the Hanácká Mana variety.

The sum of the fatty acid peak areas was 6.1 times greater in the stems of the Unicum variety than in the Hanácká Mana variety. In other parts, the values for the Unicum variety were only slightly higher (1.08 times in leaves, 1.12 times in roots, and 1.15 times in seeds).

The sum of the peak areas of phytosterols was 3.8 times greater in the stems of the Unicum variety than in the Hanácká Mana variety. In the other parts, the values for the Hanácká Mana variety were higher (1.16 times in the roots, 1.45 times in the leaves, and 1.14 times in the seeds).

In the case of carboxylic acids, the Unicum variety had 4.9 times more peak area of these substances in the leaves, 2.8 times in the stem and 1.8 times more in the grains than the Hanácká Mana variety. Only in the roots, 1.8 times larger peak areas of carboxylic acids were determined for the Hanácká Mana variety.

### 3.1. Saccharides

The main saccharide of all parts of the millet plant was sucrose, the largest peak area of which was determined in stems, followed by seeds, roots and leaves (Table 3). Another dominant saccharide in the vegetative parts of the millet plant was talose, and in the seeds, it was talopyranose. Other more abundant sugars in roots, stems, and leaves were fructose and fructopyranose. Arabitol and psicofuranose dominated the seeds.

For most individual carbohydrates, their peak areas were similar in both varieties (Figure S2). The Unicum variety had a larger peak area in glucofuranose and talose in stems, psicofuranose and fructose in leaves, and trehalose in millet seeds than the Hanácká Mana variety. Hanácká Mana had a larger peak area of psicose and sucrose in millet stems and fructose in millet leaves than the Unicum variety.

### 3.2. Amino Acids

Essential amino acids (isoleucine, phenylalanine, threonine, and valine) comprise the largest share of identified amino acids in plant roots (share of the sum of essential peak areas from all amino acids is 59.7%), followed by leaves (47.3%), stems (44.7%) and seeds (36.6%). Of the essential amino acids, valine and isoleucine had the largest peak area in millet (Table 4), namely in roots and stems. In leaves and seeds, threonine was dominant, followed by phenylalanine. The largest peak area of isoleucine and valine was determined in roots, and threonine and phenylalanine in leaves. Of the other amino acids, proline and alanine were dominant in stems, roots, leaves, and seeds. Serine was most abundant in roots and leaves.

When comparing the areas of the individual peaks of identified amino acids to the total area of all peaks in the two varieties of proso millet, the Hanácká Mana variety showed a higher peak area for all identified amino acids except threonine, for which the peak area was very similar in millet roots (Figure S3). Conversely, in stems and leaves, the peak areas of individual amino acids were significantly higher in the Unicum variety. Seeds of the Unicum variety were also richer in identified amino acids, except for threonine. There was a noticeable difference in the content of amino acids between the varieties.

### 3.3. Carboxylic Acids

Overall, the largest peak area of carboxylic acids was determined in the roots. The carboxylic acid with the largest peak area was succinic acid in millet roots, pyroglutamic acid in stems and seeds, and malic acid in leaves (Table 5). Cinnamic acid was most abundant in the roots while it was below the detection limit in seeds and stems. When comparing the varieties, it is evident that the Unicum variety had a larger area of carboxylic acid peaks in the leaves and stems of millet (Figure S4). On the contrary, the roots were richer in carboxylic acids in the Hanácká Mana variety, except for succinic acid, where the peak area was smaller than in the Unicum variety. The seeds of the Unicum variety contained succinic, malic, and fumaric acids, which, on the other hand, were below the level of detection in the seeds of the Hanácká Mana variety. Cinnamic acid was identified only in the leaves and roots of the Hanácká Mana variety.

### 3.4. Fatty Acids

Saturated fatty acids were dominant in all parts of millet, where the largest proportion of fatty acids from the peak area was in stems and leaves with 91%, followed by roots with 77% and seeds with 56%. Of the saturated fatty acids, palmitic and stearic acids were dominant in the roots and stems, while octadecenoic and palmitic acids were dominant in the leaves and seeds (Table 6). Lauric acid was identified only in millet leaves. Hexacosanoic acid was identified only in millet leaves and roots. The stems and leaves were then rich in tetradecanoic acid. Unsaturated fatty acids comprise the largest % of the peak area of the fatty acids in seeds with 40%, followed by roots with 20%, and then stems and leaves with 9%. Of the unsaturated fatty acids, only linoleic acid was identified. Fatty acid esters accounted for the largest proportion in seeds (4%) and roots (3%). Two fatty acid esters have been identified in millet plants. 1-Glyceryl mono-eicosanoate-2TMS was mainly represented in plant roots, and in the case of linoleic acid, 1,3-bis-(O-TMS)-2-propyl ester, the largest peak area was determined in millet seeds.

The content of fatty acids in the roots was balanced in both the monitored varieties. Tetradecanoic acid was additionally identified in the Unicum variety, while in the Hanácká Mana variety, linoleic acid ester, 1.3-bis-(O-TMS)-2-propyl ester was identified (Figure S5). In the stems, a larger peak area for all identified fatty acids was found in the Unicum variety. On the other hand, the areas of individual fatty acids in the leaves were larger in Hanácká Mana, except for tetracosanoic and tetradecanoic acids. In addition to tetracosanoic and tetradecanoic acids, the presence of eicosanoic acid, and a 9.6 times larger area of stearic acid than in the Hanácká Mana variety was found in the seeds of the Unicum variety.

### 3.5. Amyrin, Phytosterols and Miliacin

Amyrin was identified only in millet leaves (Table 7). Campesterol was most abundant in the roots and leaves of millet, followed by the seeds, and the smallest peak area was found in the stems. Miliacin was most abundant in millet seeds, followed by leaves, roots, and stems. β-Sitosterol was most abundant in leaves and seeds, followed by roots, and the smallest peak area was found in stems. Stigmasterol was most abundant in roots, followed by leaves, stems, and seeds. Germanicol was identified with a borderline Match Factor in leaves with a peak area 1.31 ± 0.167, and in seeds with a peak area 0.19 ± 0.214.

A larger area of the amyrin peak was found in the Hanácká Mana variety (Figure S6). Additionally, the content of miliacin in both seeds and leaves was also higher in Hanácká Mana. Campesterol was found only in the seeds of the Unicum variety. Stigmasterol had a larger peak area in the seeds and stems of the Unicum variety, while the area in the leaves was larger in the Hanácká Mana variety. The roots, leaves, and seeds of the Hanácká Mana variety were richer in β-sitosterol. On the contrary, the stems were richer in β-sitosterol in the Unicum variety.

### 3.6. Miscellaneous Compounds

Among other compounds, retinal was identified in the roots, stems, and leaves of millet (Table 8). Squalene was determined only in millet seeds. Tetramethyl-2-hexadecenol was determined only in leaves and stems. Tocopherols were present in leaves and roots of millet.

Squalene, and tocopherols had larger peak areas in the Hanácká Mana variety (Figure S7). Tetramethyl-2-hexadecenol was identified only in stems of the Unicum variety. Retinal had larger peak areas in the Unicum variety.

## 4. Discussion

### 4.1. Saccharides

Saccharides have a crucial role in the life of plants because sucrose is the major assimilate, and they are the structural and storage substances, substrates, and metabolites of many biochemical processes. The concentration in individual parts changes during the life of the plant, and their deficiency causes senescence in roots and seeds as a storage product for forming polysaccharides. The place of sugar accumulation is genetically determined [21]. The lowest content in seeds can be explained by the conversion of saccharides into storage polysaccharides.

The dominant saccharides in all parts of the millet plant were sucrose, fructose and psicose. On the contrary, turanose, trehalose, glucose and cellobiose were the least represented sugars. According to Salerno and Curatti [22], in nature, trehalose and sucrose are the sugars synthesized by similar pathways and are the most common naturally occurring disaccharides. Trehalose is found in a wide range of organisms including higher plants. In contrast, sucrose is the essential sugar in the life of higher plants, as it is the primary product of photosynthesis necessary for growth, development, and acclimation to environmental stress. Fructose is found in many plants, where it can be bonded to glucose and form sucrose. For commercial purposes, fructose is obtained, among other things, from corn, a plant from the Poaceae family [23]. This is probably the reason for the higher content of this carbohydrate in millet as well. According to Oshima et al. [24], D-psicose is a C-- epimer of D-fructose, which is found in minimal quantities in nature. However, D-psicose may be useful as a sweetening agent to reduce caloric intake.

Gonzali et al. [25] state that turanose, a non-metabolizable sucrose analog, belongs to sugars acting as signaling molecules that regulate many developmental processes in plants, including lateral and adventitious root production.

Cellobiose is the primary product of cellulose hydrolysis and is expected to function as a type of pathogen/damage-associated molecular pattern in evoking plant innate immunity [26]. We identified this compound only in roots and stems.

Of the sugar alcohols in millet, glucitol was the most represented, followed by arabitol, and erythrithol. Arabitol is used in the food industry as a sweetener and in the production of human therapeutics as an anticariogenic agent. This polyol can be produced by yeasts from agricultural waste materials [27]. Richardson et al. [28] observed that arabitol was identified in sheaths of endophyte-infected grasses, and arabitol accumulated in infected grasses under drought stress. Erythritol is a naturally occurring four-carbon sugar alcohol (polyol) that is found in a variety of fruits, such as melon, watermelon, pears, and grapes. It is used in the food and pharmaceutical industries as a natural sweetener [29]. In this study, erythritol was identified in all millet plant parts with the largest peak area in stems. Sorbitol was identified in all parts of millet plants with the highest content in leaves and roots. According to Jain et al. [30], sorbitol is a sugar alcohol biosynthesized in the cytosol of photosynthetically active plant leaves. Sorbitol accumulation is considered an adaptive response of plants to drought, salinity, or chilling stress. Metabolic utilization of sorbitol is restricted to sink plant tissues. Angiosperm plant species from Rosaceae and Plantaginaceae families, which utilize sorbitol as main photosynthetic products, are denoted as “usual sorbitol producers,” while other species producing lower amounts of sorbitol are denoted as “non-usual sorbitol producers”, which include, among others, plants from Poales, which includes millet [31].

According to our results, we can include glucofuranose among the significantly represented sugars in the vegetative parts of the millet plant (roots, leaves, and stems). According to Alexandersson and Nestor [32], glucofuranoses are extremely rare as building blocks in biomolecules. A few studies have reported glucofuranose residues from a plant polysaccharide and it is still unclear whether glucofuranose is at all present as a component of biomolecules.

### 4.2. Amino Acids

Amino acids are the building blocks for proteins and substrates of secondary metabolites, and they play many roles in plants, acting as signal molecules, regulating plant architecture, flowering, and stress defense. Amino acid accumulation is influenced by the plant species and its growth stage. Amino acids can be stored in the leaves and stem nodes before being distributed to grains. Amino acids imported into the embryo are used to synthesize storage protein. Their distribution in plants starts in the root xylem [33]. This may be the reason for the largest determined area of amino acid peaks in millet roots.

Identified essential amino acids (isoleucine, phenylalanine, threonine, and valine) comprise 36.6% share of the identified amino acids in millet seeds. Essential amino acids are amino acids that humans and other vertebrates cannot synthesize from metabolic intermediates. There are nine essential amino acids, including phenylalanine, valine, tryptophan, threonine, isoleucine, methionine, histidine, leucine, and lysine [34]. Kalinová and Moudrý [35] demonstrated apparent differences between millet varieties in lysine, valine, isoleucine, phenylalanine, and aspartic acid content. In our case, serine, phenylalanine, and glutamine were identified only in the Unicum variety, so we can assume varietal variability here. Kalinová and Moudrý [35] found via chromatographic analysis after acid and oxidative acid hydrolysis, that glutamic acid had the highest content in seeds (19 g/16 g N), and the next dominant amino acids were leucine and aspartic acid. Of the amino acids we identified, threonine, proline, and alanine had the largest peak area in millet seeds. According to Wiedemair et al. [36], who analyzed millet grains using acidic hydrolysis and ion-exchange chromatography with ninhydrin derivatization and subsequent detection with photometry, glutamic acid/glutamine (2.13  ±  0.34 g per 100 g), alanine (1.06  ±  0.18 g per 100 g) and leucine (1.36  ±  0.24 g per 100 g) are the most abundant amino acids in proso millet grains. Different extraction methods may have resulted in the different contents of the mentioned amino acids. In millet leaves, Edgar and Draper [37], using chromatographic analysis after acid hydrolysis found glutamic acid (10.08 µmol/100 µmol) to be dominant, followed by leucine (9.69 µmol/100 µmol total amino acids), asparagine (9.26 µmol/100 µmol total amino acids), and aspartic 9.26 (µmol/100 µmol total amino acids), threonine (5.41 µmol/100 µmol total amino acids), serine (6.38 µmol/100 µmol total amino acids), valine (5.50 µmol/100 µmol total amino acids), isoleucine (4.80 µmol/100 µmol total amino acids), alanine (8.66 µmol/100 µmol total amino acids), proline (5.12 µmol/100 µmol total amino acids), and phenylalanine (4.66 µmol/100 µmol total amino acids). Of the amino acids we identified in millet leaves, threonine, proline, and alanine had the largest peak area. In roots of 21-day-old millet plant, Edgar and Draper [37] determined the highest content of aspartic acid (18.13 µmol/100 µmol total amino acids) and glutamic acid (17.49 µmol/100 µmol total amino acids) followed by serine (10.3 µmol/100 µmol total amino acids), alanine (9.7 µmol/100 µmol total amino acids), proline (3.79 µmol/100 µmol total amino acids), valine (3.57 µmol/100 µmol total amino acids), threonine (3.99 µmol/100 µmol total amino acids), and isoleucine (1.78 µmol/100 µmol total amino acids). Of the amino acids we identified in the millet roots, isoleucine, valine, proline, alanine, and threonine had the largest peak area.

### 4.3. Carboxylic Acids

Carboxylic acids are frequently detected in root exudates [38], which can probably be the reason for their higher level in roots. Pyroglutamic acid, also known as 5-oxoproline pyrrolidone 2-carboxylic acid, is a cyclic lactam of glutamic acid and becomes a metabolite in the glutathione cycle. It is also a constrained analog of gamma-aminobutyric acid that is considered a physiological moisturizer because it is associated with acting on the intracellular and extracellular water flow, predisposing it, among other things, to wide use in cosmetics (Mejri et al. 2019) [39]. Their participation in the water cycle of the plant is probably the reason why it was identified in all parts of the millet plant and was the largest representation of the identified carboxylic acids.

Succinic acid, a four-carbon dicarboxylic acid, was the second most abundant carboxylic acid in millet plants. The reason for its abundant presence in all parts of plants is that it plays an important role in metabolic processes, such as the synthesis and breakdown of saccharides and fatty acids. In all parts of the millet plants, the areas of the succinic acid peak were larger in the Unicum variety, so varietal variability in the content of this substance can be assumed.

Malic acid is one of the most widely distributed organic acids found in plants and frequently occurs in relatively high concentrations [40]. The presence of malic acid (18.89 ± 2.77 g/L) in millet is mentioned in the literature together with oxalic acid (1.94 ± 0.01 g/L), tartaric acid (8.31 ± 0.15 g/L), pyruvic acid (2.48 ± 77 g/L), and succinic acid (135.76 ± 1.243 g/L) in HPLC analyzed grains after their soaking and boiling before the start of fermentation in the preparation of traditional alcoholic Chinese beverage [41]. The largest peak area of malic acid was measured by us in roots and stems, followed by leaves, and the smallest amount was detected in seeds. According to Endo and Ikusima [42], the concentration of malic acid in the green parts is affected by the fixation of CO_2_ when the accumulation of malic acid in leaves in the dark and the release of CO_2_ accompanied by the decomposition of malic acid in the light.

Fumaric acid is a naturally occurring four-carbon dicarboxylic acid, an intermediate in the citric acid cycle that is widely distributed in plants. Fumaric acid is a valuable compound used in foods, beverages, detergents, animal feed, pharmaceuticals, and other industrial products [43]. Fumaric acid can be metabolized to yield energy and carbon skeletons to produce other compounds [44]. Information on fumaric acid content of millet is scarce. Lima et al. [45] reported trace amounts of fumaric acid in millet seeds using ultra-fast liquid chromatography. In this study, we identified fumaric acid in roots and leaves in addition to seeds. Chia et al. [44] state that fumaric acid concentrations increased with plant age and light intensity in Arabidopsis leaves and Arabidopsis phloem exudates also contained significant quantities of fumaric acid. The content of fumaric acid in the exudates may be the reason for the presence of fumaric acid in the stems and roots of millet that we determined.

Cinnamic acid is one of the aromatic carboxylic acids widely represented in plants. It shows antioxidant, antimicrobial, anticancer, neuroprotective, anti-inflammatory, and antidiabetic activities [46]. Cinnamic acid is also a known allelochemical that affects seed germination and plant root growth and therefore influences several metabolic processes [47]. Jeon et al. [48] determined the content of cinnamic acid to be in the range of 3.45 to 9.82 μg/g on the dry matter in the seeds of different millet varieties using gas chromatography. We found this acid in the leaves and roots; in the seeds, it was below the detection limit.

### 4.4. Fatty Acids

Fatty acids are used for the synthesis of plastidial and other cellular membranes in all plant cells. In certain plant tissues, most notably in seeds, they are used to synthetize storage lipids [49]. Lipids are minor constituents in cereal grains. Their content in dehulled seeds of millet ranges from 3.5 to 6.7% [50]. Lorenz and Hwang [51] determined the lipid composition of flours and brans of nine proso millet varieties using gas chromatography. They reported the highest representation of linoleic acid (a polyunsaturated omega-6 fatty acid), followed by oleic acid (a monounsaturated omega-9 fatty acid), palmitic acid (saturated fatty acid), palmitoleic acid (an omega-7 monounsaturated fatty acid), octadecanoic acid (stearic, saturated fatty acid), α-linolenic acid (a polyunsaturated omega-3 fatty acid), arachidonic acid (polyunsaturated omega-6 fatty acid) and hexadecenoic acid (palmitoleic acid, monounsaturated fatty acid). Lima et al. [45] determined the following fatty acids in millet seeds using gas chromatography with flame ionization detection. The acids are listed in decreasing order: oleic acid, palmitic acid, linoleic acid, heptadecanoic acid, stearic acid, caproic acid, caprylic acid, capric acid, and myristic acid. We identified the unsaturated fatty acid linoleic acid as the main representative of fatty acids in millet seeds, followed by the saturated fatty acids palmitic acid, octadecenoic acid, stearic acid, eicosanoic acid, tetradecanoic acid (myristic acid), and tetracosanoic acid. We have confirmed that fatty acids have a significant presence in other parts as well; in leaves, stems, and roots of millet.

### 4.5. Phytosterols, Amyrin, and Miliacin

Amyrin is a pentacyclic triterpenol that is widely distributed in the epicuticular waxes of angiosperm plants. Tulloch (1982) [52] identified α- and β-amyrin in epicuticular waxes of millet leaves using GC–MS, where α-amyrin constituted 40% of esterified triterpene alcohols and β-amyrin 18%. Our detection of amyrin in the leaves also corresponds to this. In contrast, Bossard et al. [53] determined that the concentration of amyrin in individual parts of millet and the total concentration of amyrin decreased from the stem (12.5 µg/g), through the seeds (11 µ/g) to the leaves (9.7 µg/g) using GC–MS. Their presence in other plant parts, such as stems and seeds, is because they also contain epicuticular waxes. In our study, 2.3 times larger amyrin peak area was detected in the leaves of the Hanácká Mana variety, which can be explained by the appearance of their leaves which are rougher, darker, and thicker than the Unicum variety.

Miliacin (olean-18-en-3β-ol methyl ether) belongs to pentacyclic triterpenes, and it is an important raw material for medicine, food industry, or cosmetics, especially due to the preventive effect of millet on hair loss [54]. We found miliacin in all vegetative parts of millet (roots, stems, and leaves) and in seeds. In seeds, the miliacin peak area was 1.3 times larger, and in leaves, it was 1.5 times larger in the Hanácká Mana variety than in the Unicum variety. On the other hand, in stems, the miliacin peak area was 2.8 times larger in the Unicum variety than in the Hanácká Mana variety. Similarly, An et al. [54] reported significant differences in the miliacin content in seeds of different millet varieties. According to Bossard et al. [53], the concentration of milliacin in individual parts of millet, determined via GC–MS, decreased from seeds (306 μg/g) through stems (171 μg/g) and roots (23.9 μg/g) to leaves (7.5 μg/g). In our case, the largest peak area was in seeds, followed by leaves and it was the same level in roots and stems.

Phytosterols, such as β-sitosterol, campesterol, and stigmasterol are components of plant cell membranes and have a broad spectrum of biological effects, including anti-inflammatory, antioxidative, and anticarcinogenic activities [55]. We found campesterol, stigmasterol, and β-sitosterol in all plant parts (roots, stems, leaves, and seeds). Ryan et al. [55], using HPLC, determined 48.3 mg/100 g of β- sitosterol, 8.7 mg/100 g of campesterol and 0.8 mg/100 g of stigmasterol in millet seeds. This is probably the first data on the presence of these sterols in other parts of the millet plant. From the results of our work, varietal variability in the content of these substances can also be assumed, but the validity of this assumption needs further verification. In this study, another pentacyclic triterpenoid, namely germanicol, was found which had the largest peak area in leaves and a smaller peak area in millet seeds Its presence in millet seeds was also confirmed by Bossard et al. [53], who quantified, using GC–MS, germanicol in leaves, stems, and seeds of the Sunrise variety, wherein he also found the highest content in leaves (30.5 μg/g), followed by stems (8.3 μg/g) and seeds (6.6 μg/g).

### 4.6. Miscellaneous Compounds

Squalene is a triterpene with six isoprene units known as a natural antioxidant in several plants, such as amaranth or buckwheat [48]. Unlike buckwheat, where squalene was identified in the vegetative parts of the plant in addition to the seeds, the presence of squalene in millet was identified only in the seeds. The presence of squalene in millet was also confirmed by Ryan et al. [56], who determined 8.8 mg/100 g of squalene in seeds using HPLC.

According to Dickinson et al. [57], carotenoids are precursors of plant hormones strigolactones and abscisic acid, as well as multiple bioactive molecules and several endogenous carotenoid-derived metabolites are identified as new regulators of root growth and development in plants, among them also retinal. Retinal (vitamin A aldehyde) binding precedes the root clock and predicts sites of lateral root organogenesis. According to Ke et al. [58], retinal could be produced from the oxidative cleavage of β-carotene. However, it lacks experimental evidence. Proso millet is known for the high content of carotenoids in its seeds. Kim et al. [59] determined the concentration of β-carotene in millet seed to be between 0.04 and 0.06 ug/g DW depending on the variety. This is probably the first data on retinal presence in millet plants, where retinal was identified in all vegetative parts with the largest area of peak in the roots. In all parts of the millet plant, the retinal area was higher in the Unicum variety, which is more sensitive to environmental conditions than the Hanácká Mana variety. Varietal variability in retinol content can therefore be assumed, mainly with the variety’s resistance to environmental conditions. However, this assumption must be verified experimentally.

Tocopherols include four isoforms (α, β, γ, and δ) that together with tocotrienols form the vitamin E family. Lima et al. [45] detected all tocopherol isoforms in millet seeds using HPLC, and γ-tocopherol was the most abundant (3.00 ± 0.03 mg/100 g DW). However, tocopherols are also present in vegetative plant parts [48]. We confirmed the presence of tocopherols in vegetative plant parts of millet when tocopherols were identified in the leaves and the roots of millet. The total tocopherols in the seeds were below the detection limit.

Tetramethyl-2-hexadecenol was identified in the green parts of millet, leaves and stems. According to Patton and Benson [60], 3,7,11,15-tetramethyl 2-hexadecenol (phytol) is a precursor of the branched-chain fatty acid with biological significance, 3,7,11,15-tetramethyl hexadecanoic acid, and play an important role in positioning the chlorophyll molecule in the chloroplast membrane [61]. 3,7,11,15-tetramethyl-2-hexadecen-1-ol was determined in buckwheat sprouts (11–15%) using via GC–MS and belonged to the major volatile components [62]. The presence of this compound was probably recorded in millet stems for the first time and only in the Unicum variety; on the contrary, the Hanácká Mana variety had a larger peak area in the millet leaves, which may be related to the length of the growing season of the varieties (growing season for the Unicum variety is shorter than the Hanácká Mana variety), or the height of the plants (Hanácká Mana is a taller variety than the Unicum variety). However, this assumption needs further experimental verification.

## 5. Conclusions

For analysis or screening of substances in all plant parts of the two proso millet varieties, a metabolomic approach was used, and all important primary metabolites (amino acids, sugars, and carboxylic acids) and selected secondary metabolites were searched for and analyzed. This procedure made it possible, on one hand, to study the changes in the content of metabolites related to the structure of millet plants and the influence of the external environmental conditions, and on the other hand, to describe substances that, based on our knowledge, are described in millet plants for the first time, e.g., retinal.

Unlike previous studies, this study brings new information about the distribution of the known, newly identified substances or substances whose presence has been described so far only in millet grain, in different plant parts of proso millet.

## Figures and Tables

**Table 1:** Temperature gradient of gas chromatography.

GC Oven Number	Rate (°C per min)	Temperature (°C)	Hold Time (min)
Initial temperature	0	60	1
1	40	200	0
2	5	290	20

**Table 2:** The sum of the peak areas of the identified main groups of substances expressed in % of the total area of all peaks in proso millet (mean values for varieties Unicum and Hanácká Mana).

Compound	Plant Part			
	Roots	Stems	Leaves	Seeds
Saccharides	71.33 ab	82.75 b	53.12 ab	46.16 a
Amino acids	6.96	2.54	4.68	1.81
Fatty acids	11.17	8.41	14.68	24.60
Carboxylic acids	3.00	1.92	1.34	1.36
Phytosterols	2.77 a	0.92 a	4.29 a	10.51 b
Miscellaneous	1.39	1.39	4.14	1.29
Unidentified	1.04	0.00	5.79	0.19

Different letters (a,b) in the column represent statistically significant differences (*p* < 0.05) among parts.

**Table 3:** Proportional representation of the areas of individual peaks of identified saccharides to the total area of all peaks in % [mean for varieties Unicum and Hanácká Mana ± SD].

Compound	Plant Part			
	Roots	Stems	Leaves	Seeds
**Monosaccharides**				
Fructopyranose	5.05 ± 0.294 c	3.85 ± 0.237 bc	3.19 ± 0.237 b	0.35 ± 0.398 a
Fructose	8.54 ± 0.525	3.31 ± 2.373	4.45 ± 2.003	0.95 ± 0.393
Fucopyranose/Fucose	0.11 ± 0.127 a	n.d. a	2.01 ± 0.277 b	n.d. a
Glucofuranose	4.56 ± 0.219	4.73 ± 3.741	2.37 ± 1.484	0.13 ± 0.134
Glucose	n.d. a	0.64 ± 0.231 b	0.09 ± 0.098 ab	n.d. a
Psicofuranose	n.d.	n.d.	1.74 ± 2.003	1.37 ± 0.548
Psicose	3.94 ± 0.110	4.29 ± 4.295	2.52 ± 0.416	n.d.
Rhamnopyranose	0.05 ± 0.052 a	0.01 ± 0.012 a	1.73 ± 0.219 b	n.d. a
Talopyranose	6.07 ± 0.225	4.39 ± 1.518	3.85 ± 0.081	2.52 ± 0.323
Talose	8.42 ± 0.473	6.24 ± 4.971	5.75 ± 0.058	1.85 ± 1.975
**Disaccharides**				
Cellobiose	0.36 ± 0.046 b	0.10 ± 0.115 ab	n.d. a	n.d. a
Trehalose	0.40 ± 0.052	n.d.	0.79 ± 0.231	0.82 ± 0.947
Sucrose	28.23 ± 4.163	51.25 ± 22.430	18.74 ± 0.139	35.36 ± 5.196
Turanose	0.06 ± 0.069	0.19 ± 0.219	n.d.	0.14 ± 0.162
**Sugar alcohols and others**				
Arabitol	1.39 ± 0.323	n.d.	1.86 ± 0.803	1.88 ± 0.491
Erythritol	0.24 ± 0.104	1.02 ± 0.214	0.65 ± 0.219	0.15 ± 0.153
Sorbitol	3.17 ± 0.075 b	2.05 ± 0.439 ab	2.99 ± 0.335 b	0.57 ± 0.658 a
Threonic acid	0.78 ± 0.433	0.30 ± 0.179	0.42 ± 0.035	0.09 ± 0.098

Different letters (a–c) in the column represent statistically significant differences (*p* < 0.05) among parts.; n.d.—below the detection limit.

**Table 4:** Proportional representation of the areas of individual peaks of identified amino acids to the total area of all peaks in % [mean for varieties Unicum and Hanácká Mana ± SD].

Compound	Plant Part			
	Roots	Stems	Leaves	Seeds
**Isoleucine**	1.86 ± 1.128	0.45 ± 0.381	0.44 ± 0.162	n.d.
**Phenylalanine**	n.d.	n.d.	0.57 ± 0.562	0.14 ± 0.156
**Threonine**	0.44 ± 0.029 ab	0.24 ± 0.144 a	0.77 ± 0.087 b	0.53 ± 0.040 ab
**Valine**	1.86 ± 1. 828	0.45 ± 0.381	0.44 ± 0.162	n.d.
Alanine	0.99 ± 0.237	0.56 ± 0.358	0.89 ± 0.289	0.42 ± 0.012
Glutamine	n.d.	n.d.	0.57 ± 0.652	0.14 ± 0.162
Proline	1.58 ± 0.208	0.74 ± 0.491	0.79 ± 0.191	0.52 ± 0.075
Serine	0.24 ± 0.023	0.11 ± 0.069	0.23 ± 0.133	0.07 ± 0.081

Different letters (a,b) in the column represent statistically significant differences (*p* < 0.05) among parts; n.d.—below the detection limit.

**Table 5:** Proportional representation of the areas of individual peaks of identified carboxylic acids to the total area of all peaks in % [mean for varieties Unicum and Hanácká Mana ± SD].

Compound	Plant Part			
	Roots	Stems	Leaves	Seeds
Cinnamic acid	0.57 ± 0.562	n.d.	0.13 ± 0.144	n.d.
Fumaric acid	0.17 ± 0.191	0.15 ± 0.167	n.d.	0.09 ± 0.098
Malic acid	0.70 ± 0.283	0.67 ± 0.346	0.51 ± 0.214	0.10 ± 0.115
Pyroglutamic acid	0.72 ± 0.115	0.70 ± 0.427	0.42 ± 0.485	1.04 ± 0.064
Succinic acid	0.86 ± 0.248	0.41 ± 0.121	0.41 ± 0.323	0.14 ± 0.156

No statistically significant differences (*p* < 0.05) were among parts; n.d.—below the detection limit.

**Table 6:** Proportional representation of the areas of individual peaks of identified fatty acids to the total area of all peaks in % [mean for varieties Unicum and Hanácká Mana ± SD].

Compound	Plant Part			
	Roots	Stems	Leaves	Seeds
**Saturated acids**				
Eicosanoic acid	0.56 ± 0.046	0.22 ± 0.075	0.60 ± 0.092	0.24 ± 0.231
Hexacosanoic acid	0.19 ± 0.023 b	n.d. a	0.15 ± 0.012 b	n.d. a
Lauric acid	n.d. a	n.d. a	1.01 ± 0.318 b	n.d. a
Octadecenoic acid	1.20 ± 0.069 a	0.60 ± 0.329 a	2.82 ± 0.254 b	5.07 ± 0.439 c
Palmitic acid	2.42 ± 0.179 a	1.07 ± 0.566 a	2.51 ± 0.214 a	5.76 ± 0.058 b
Stearic acid	2.13 ± 0.017	1.07 ± 0.537	2.13 ± 0.127	2.38 ± 2.229
Tetracosanoic acid	0.90 ± 0.001	0.36 ± 0.012	0.73 ± 0.312	0.13 ± 0.134
Tetradecanoic acid	1.18 ± 1.057	4.35 ± 4.023	3.38 ± 1.472	0.26 ± 0.294
**Unsaturated acids**				
Linoleic acid	2.25 ± 0.294 b	0.75 ± 0.439 a	1.28 ± 0.214 ab	9.84 ± 0.052 c
**Esters**				
1-Glyceryl mono-eicosanoate-2TMS	0.25 ± 0.046	n.d.	0.09 ± 0.104	n.d.
Linoleic acid, 1.3-bis-(O-TMS)-2-propyl ester	0.11 ± 0.121	n.d.	n.d.	0.94 ± 0.514

Different letters (a–c) in the column represent statistically significant differences (*p* < 0.05) among parts; n.d.—below the detection limit.

**Table 7:** Proportional representation of the areas of individual peaks of identified phytosterols, amyrin, and miliacin to the total area of all peaks in % [mean for varieties Unicum and Hanácká Mana ± SD].

Compound	Plant Part			
	Roots	Stems	Leaves	Seeds
Amyrin	n.d.	n.d.	0.95 ± 0.439	n.d.
Campesterol	0.37 ± 0.058	0.02 ± 0.023	0.37 ± 0.081	0.23 ± 0.266
Miliacin	0.19 ± 0.017 a	0.18 ± 0.098 a	0.36 ± 0.087 a	7.86 ± 1.160 b
β-Sitosterol	1.20 ± 0.162 ab	0.52 ± 0.381 a	2.62 ± 0.531 b	2.27 ± 0.069 b
Stigmasterol	1.02 ± 0.029 b	0.2 ± 0.150 a	0.95 ± 0.214 ab	0.16 ± 0.179 a

Different letters (a,b) in the column represent statistically significant differences (*p* < 0.05) among parts; n.d.—below the detection limit.

**Table 8:** Proportional representation of the areas individual peaks of identified miscellaneous compounds to the total area of all peaks in % [mean for varieties Unicum and Hanácká Mana ± SD].

Compound	Plant Part			
	Roots	Stems	Leaves	Seeds
Retinal	1.30 ± 0.947	0.26 ± 0.173	0.16 ± 0.023	n.d.
Squalene	n.d. a	n.d. a	n.d. a	1.29 ± 0.104 b
Tetramethyl-2-hexadecenol	n.d.	1.13 ± 1.305	1.84 ± 0.964	n.d.
Tocopherols	0.09 ± 0.104 a	n.d. a	2.15 ± 0.167 b	n.d. a

Different letters (a,b) in the column represent statistically significant differences (*p* < 0.05) among parts; n.d.—below the detection limit.

## Data Availability

The data used to support the findings of this study can be made available by the corresponding author upon request.

## Supplementary Materials

The following supporting information can be downloaded at: https://www.mdpi.com/article/10.3390/foods12122294/s1, Table S1: The limits of detection (LOD) and quantification (LOQ) for internal standard (Fluoranthene-D10), Table S2: Components identified in proso millet, Figure S1. Comparison of the relative representation of the sums of the peak areas of the identified main groups of substances to the total area of all peaks (%) in Hanácká Mana and Unicum varieties of proso millet, Figure S2. Comparison of the relative representation of the areas of individual peaks of the identified saccharides to the total area of all peaks (%) in Hanácká Mana and Unicum varieties of proso millet, Figure S3. Comparison of the relative representation of the areas of individual peaks of identified amino acids to the total area of all peaks (%) in varieties Hanácká Mana and Unicum of proso millet, Figure S4. Comparison of the relative representation of the areas of individual peaks of identified carboxylic acids to the total area of all peaks (%) in varieties Hanácká Mana and Unicum of proso millet, Figure S5. Comparison of the relative representation of the areas of individual peaks of identified fatty acids to the total area of all peaks (%) in varieties Hanácká Mana and Unicum of proso millet, Figure S6. Comparison of the relative representation of the areas of individual peaks of identified phytosterols, amyrin and miliacin to the total area of all peaks (%) in varieties Hanácká Mana and Unicum of proso millet, Figure S7. Comparison of the relative representation of the areas of individual peaks of identified miscellaneous compounds to the total area of all peaks (%) in varieties Hanácká Mana and Unicum of proso millet.
