# Supplementary material for: Comparison of the Main Constituents in Two Varieties of Proso Millet Using GC–MS
Source: Foods. 2023 Jun 7;12(12):2294. doi: 10.3390/foods12122294 (PMC10297711; doi:10.3390/foods12122294)
Supplement: Supplementary file 1 [file foods-12-02294-s001.zip › foods-2402299-supplementary.pdf]

Table S1: The limits of detection (LOD) and quantification (LOQ) for internal standard (Fluoranthene-D10)

Variety	Hanácká Mana				Unicum			
Plant part	roots	stems	leaves	seeds	roots	stems	leaves	seeds
S/N	401	297	211	653	306	339	241	405
Area	20753371	25539484	30916506	28529223	22825276	30340553	28782286	22789320
LOD [ng/ml]	8.90	12.02	16.92	5.47	11.67	10.53	14.81	8.81
LOQ [ng/ml]	29.68	40.07	56.40	18.22	38.89	35.10	49.38	29.38

c(IS) = 1.19 ug/ml of extract

Table S2: Components identified in proso millet

RT (min)	Compound	Molecular Formlua	MW	MF
4.15	Alanin-2TMS	$C_9H_{23}NO_2Si_2$	233.46	919
4.64	Valin-2TMS	$C_{11}H_{27}NO_2Si_2$	261.51	916
4.82	Glycerol-3TMS	$C_{12}H_{32}O_3Si_3$	308.64	897
4.84	Phosphoric acid-3TMS	$C_{15}H_{41}O_6PSi_4$	460.80	909
4.94	Isoleucin-2TMS	$C_{12}H_{29}NO_2Si_2$	275.54	871
4.99	Proline-2TMS	$C_{11}H_{25}NO_2Si_2$	259.50	772
5.01	Succinic acid-2TMS	$C_{10}H_{22}O_4Si_2$	266.00	873
5.15	Fumaric acid-2TMS	$C_{10}H_{20}O_4Si_2$	260.44	894
5.18	Serin-3TMS	$C_{12}H_{31}NO_3Si_3$	321.64	920
5.3	Threonin-3TMS	$C_{13}H_{33}NO_3Si_3$	335.66	890
5.73	Malic acid-3TMS	$C_{13}H_{30}O_5Si_3$	350.63	931
5.8**2**	mezo-Erythritol-4TMS	$C_{16}H_{42}O_4Si_4$	410.85	919
5.98	Pyroglutamic acid-2TMS	$C_{11}H_{23}NO_3Si_2$	273.48	896
6.07	Threonic acid-4TMS	$C_{16}H_{40}O_5Si_4$	424.83	862
6.47	Rhamnopyranose-4TMS	$C_{18}H_{44}O_5Si_4$	452.88	910
6.57	Phenylalanine-2TMS	$C_{15}H_{27}NO_2Si_2$	309.55	884
6.68	Lauric acid-TMS (Dodecanoic)	$C_{15}H_{32}O_2Si$	272.50	880
6.93	Fucose/Fucopyranose-4TMS	$C_{18}H_{44}O_5Si_4$	452.88	885
7.05	Arabitol-5TMS	$C_{20}H_{52}O_5Si_5$	513.05	924
7.09	Pentahydroxycyclohexanone-5TMS	$C_{21}H_{50}O_6Si_5$	539.04	707
7.32	Phosphoric acid, 2,3-bis(trimethylsiloxy)propyl bis(trimethylsilyl) ester	$C_{15}H_{41}O_6PSi_4$	460.80	820
7.47	Glutamine-3TMS	$C_{14}H_{34}N_2O_3Si_3$	362.69	868
7.69	Fructofuranose-5TMS	$C_{21}H_{52}O_6Si_5$	541.06	923
7.74	Psicofuranose- 5TMS	$C_{21}H_{52}O_6Si_5$	541.06	900
7.87	Fructopyranose-5TMS	$C_{21}H_{52}O_6Si_5$	541.06	890
7.98	Glucofuranose-5TMS	$C_6H_{12}O_6Si_5$	320.58	< 700
8.15	Tetradecanoic acid-TMS	$C_{17}H_{36}O_2Si$	300.60	832

8.44	Psicose-5TMS	$C_{21}H_{52}O_6Si_5$	541.06	875
8.49	Talose-5TMS	$C_{21}H_{52}O_6Si_5$	541.10	904
8.87	Sorbitol-6TMS	$C_{24}H_{62}O_6Si_6$	615.26	907
8.94	Glucose-5TMS	$C_{21}H_{52}O_6Si_5$	541.06	
9.04	Cinnamic acid-2TMS	$C_{15}H_{24}O_3Si_2$	308.52	864
9.28	Talose/Talopyranose-5TMS	$C_{21}H_{52}O_6Si_5$	541.06	913
10.15	Palmitic acid-TMS	$C_{19}H_{40}O_2Si$	328.61	893
11.71	Tetramethyl-2-hexadecene-1-ol-TMS	$C_{23}H_{48}OSi$	368.71	928
12.20	Linoleic acid-TMS	$C_{21}H_{38}O_2Si$	350.61	889
12.27	Octadecenoic acid	$C_{18}H_{34}O_2$	282.50	796
12.61	Stearic acid-TMS	$C_{21}H_{44}O_2Si$	356.66	923
14.00	Retinal	$C_{20}H_{28}O$	284.44	729
15.30	Eicosanoic acid-TMS	$C_{23}H_{48}O_2Si$	384.71	852
16.43	Sucrose-8TMS	$C_{36}H_{86}O_{11}Si_8$	919.75	882
17.37	Sucrose-8TMS	$C_{36}H_{86}O_{11}Si_8$	919.75	856
18.08	Sucrose-8TMS	$C_{36}H_{86}O_{11}Si_8$	919.75	930
19.13	Turanose-8TMS	$C_{36}H_{86}O_{11}Si_8$	919.75	801
19.24	Cellobiose-8TMS	$C_{36}H_{86}O_{11}Si_8$	919.75	< 700
19.52	Trehalose-8TMS	$C_{36}H_{86}O_{11}Si_8$	919.75	913
19.62	Linoleic acid, 1,3-bis-(O-TMS)-2-propyl ester	$C_{27}H_{54}O_4Si_2$	498.9	719
19.66	Aucubin-6TMS	$C_{33}H_{70}O_9Si_6$	779.41	758
20.51	Squalene	$C_{30}H_{50}$	410.72	795
20.77	Tetracosanoic acid-TMS	$C_{27}H_{56}O_2Si$	440.80	828
22.57	1-Glyceryl mono-eicosanoate-2TMS	$C_{29}H_{62}O_4Si_2$	530.97	682
22.84	Tocopherol-TMS	$C_{32}H_{58}O_2Si$	502.90	893
23.40	Hexacosanoic acid-TMS	$C_{29}H_{60}O_2Si$	468.90	823
24.96	Tocopherol-TMS	$C_{32}H_{58}O_2Si$	502.89	912
25.06	Cholesterol-TMS	$C_{30}H_{54}OSi$	458.84	897
26.94	Campesterol-TMS	$C_{31}H_{56}OSi$	472.86	870
27.31	Miliacin	$C_{31}H_{52}O$	440.70	811
27.47	Stigmasterol-TMS	$C_{32}H_{56}OSi$	484.87	918
28.79	β-Sitosterol-TMS	$C_{32}H_{58}OSi$	486.89	901
29.07	Triacontoxy-TMS	$C_{33}H_{70}OSi$	510.99	746
29.32	Amyrin_TMS	$C_{33}H_{58}OSi$	498.90	860
29.53	Germanicol-TMS	$C_{33}H_{58}OSi$	498.90	762
31.50	probably Triacontanoic acid-TMS	$C_{33}H_{68}O_2Si$	524.98	< 700
35.12	Dotriacontyloxy-TMS	$C_{35}H_{74}OSi$	539.04	746

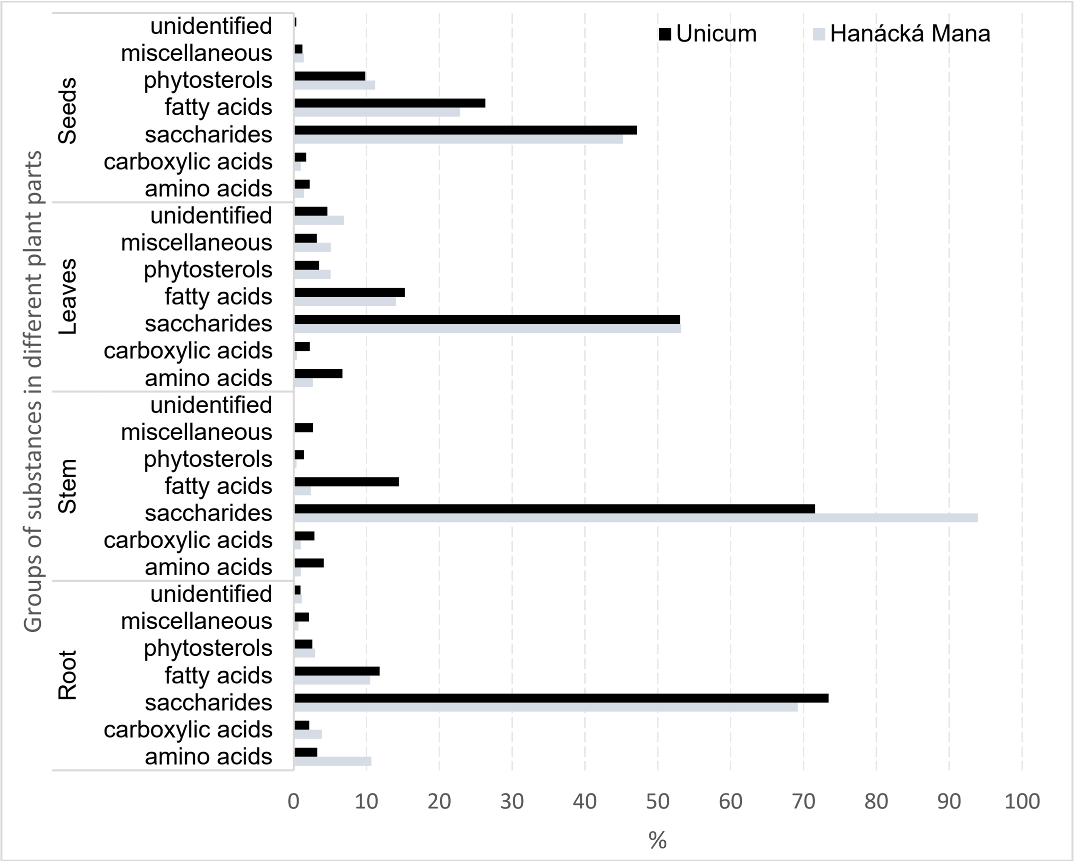

Figure S1. Comparison of the relative representation of the sums of the peak areas of the identified main groups of substances to the total area of all peaks (%) in Hanácká Mana and Unicum varieties of proso millet.

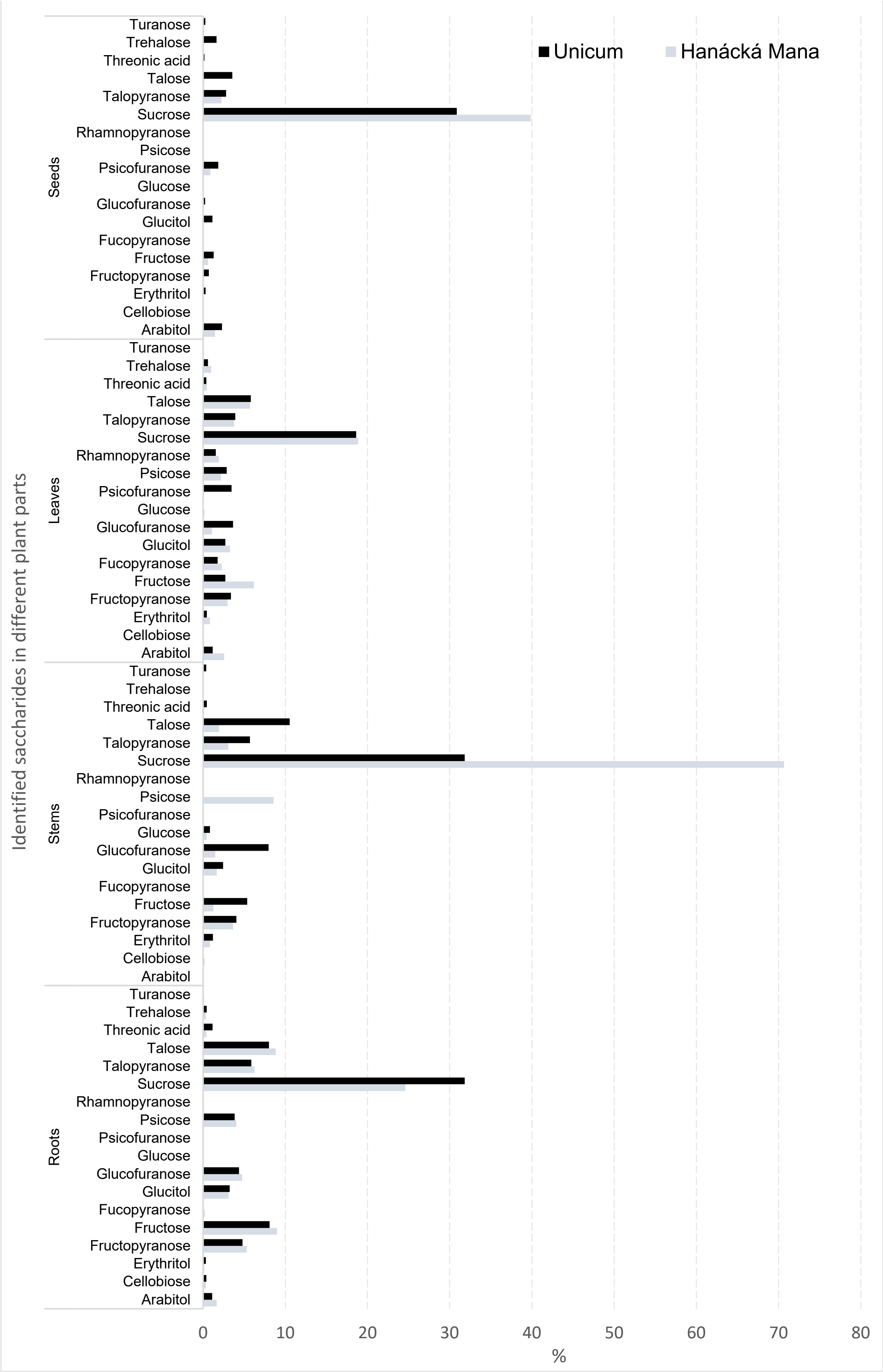

Figure S2. Comparison of the relative representation of the areas of individual peaks of the identified saccharides to the total area of all peaks (%) in Hanácká Mana and Unicum varieties of proso millet.

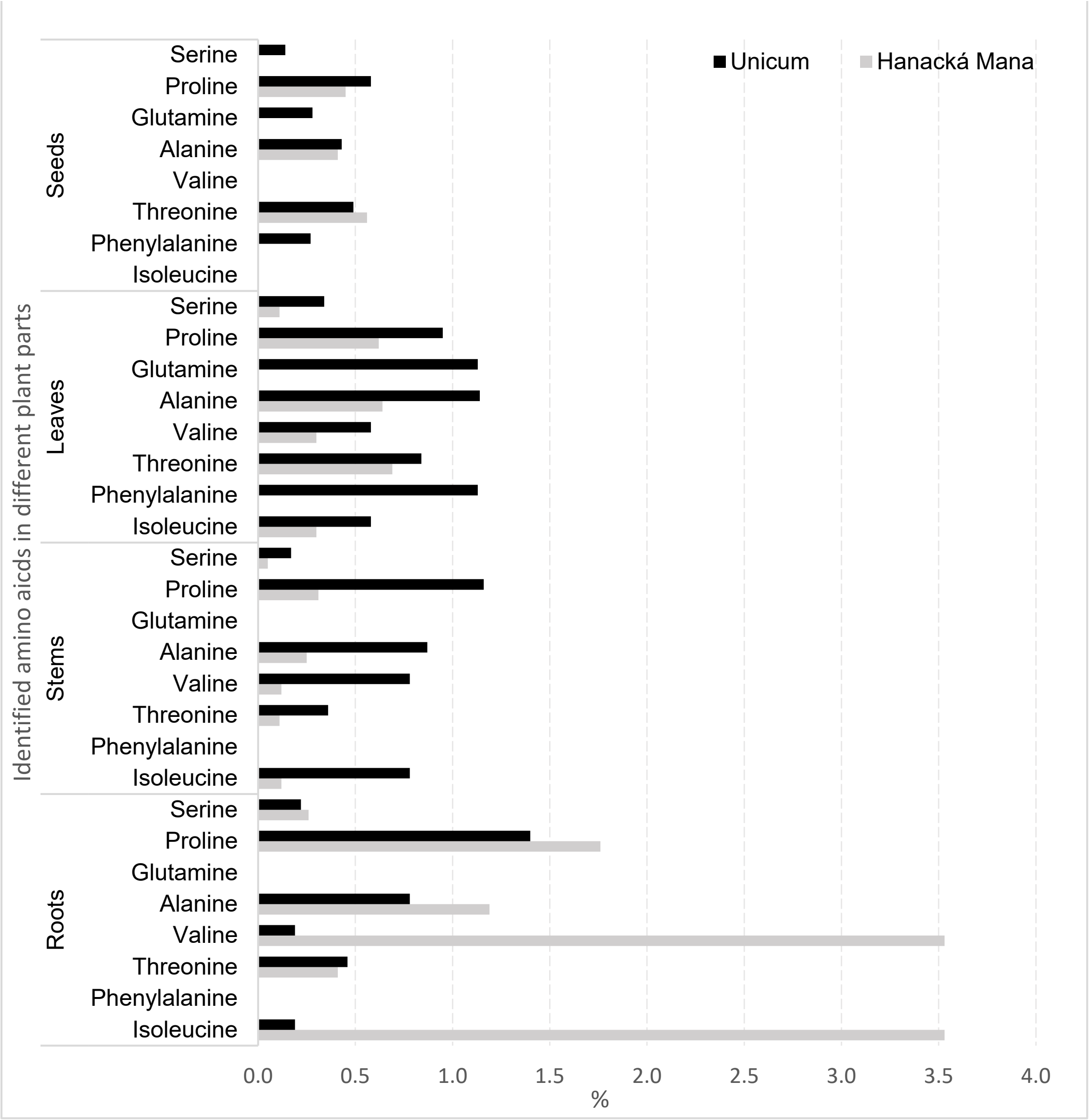

Figure S3. Comparison of the relative representation of the areas of individual peaks of identified amino acids to the total area of all peaks (%) in varieties Hanácká Mana and Unicum of proso millet.

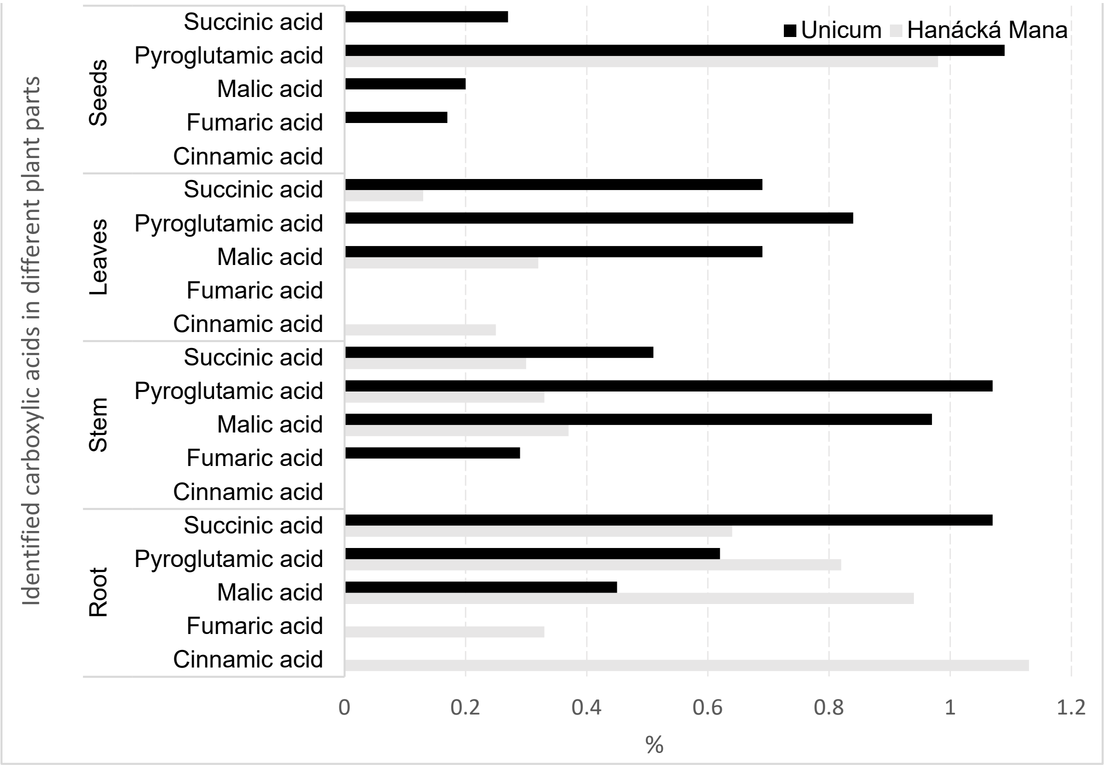

Figure S4. Comparison of the relative representation of the areas of individual peaks of identified carboxylic acids to the total area of all peaks (%) in varieties Hanácká Mana and Unicum of proso millet.

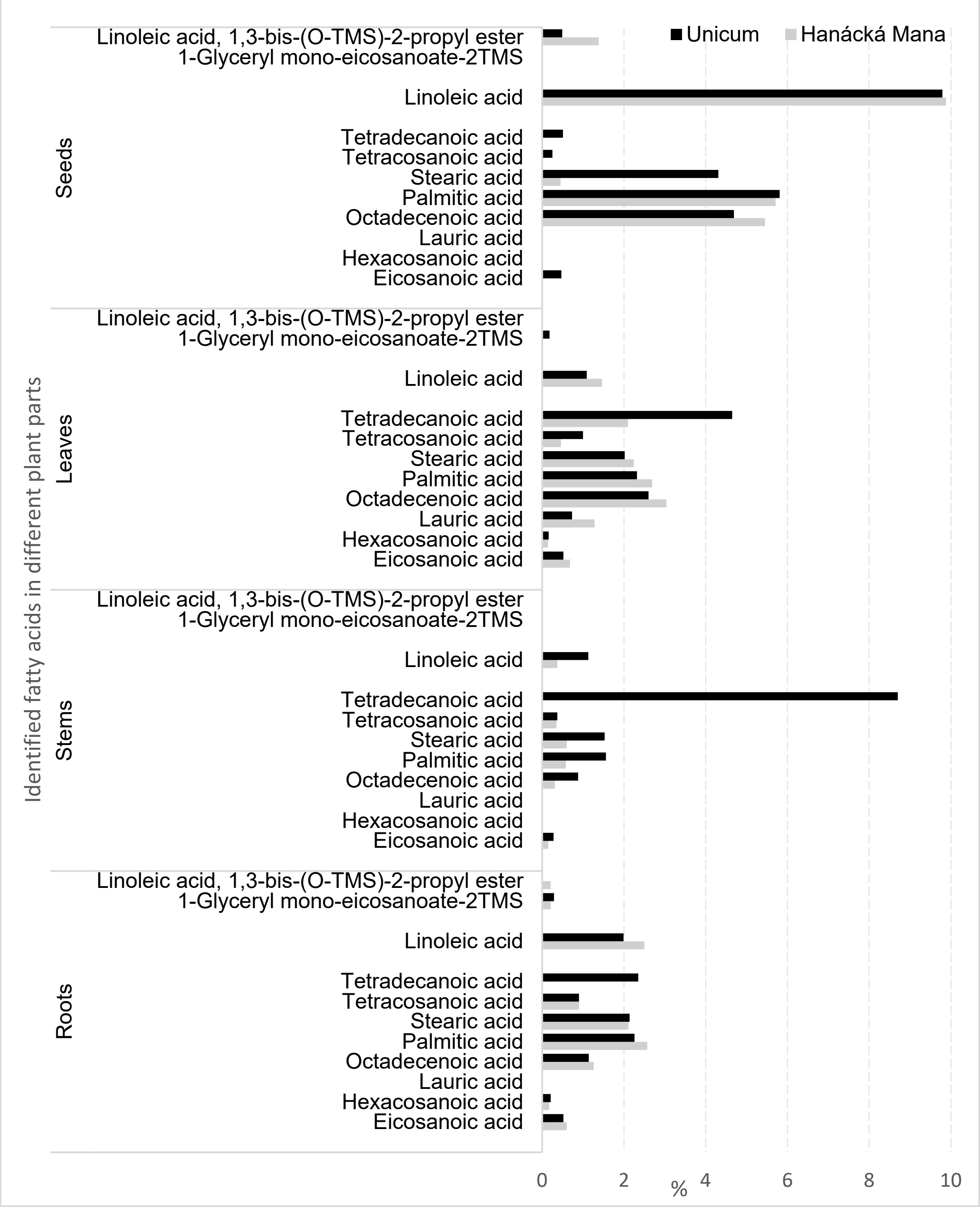

Figure S5. Comparison of the relative representation of the areas of individual peaks of identified fatty acids to the total area of all peaks (%) in varieties Hanácká Mana and Unicum of proso millet.

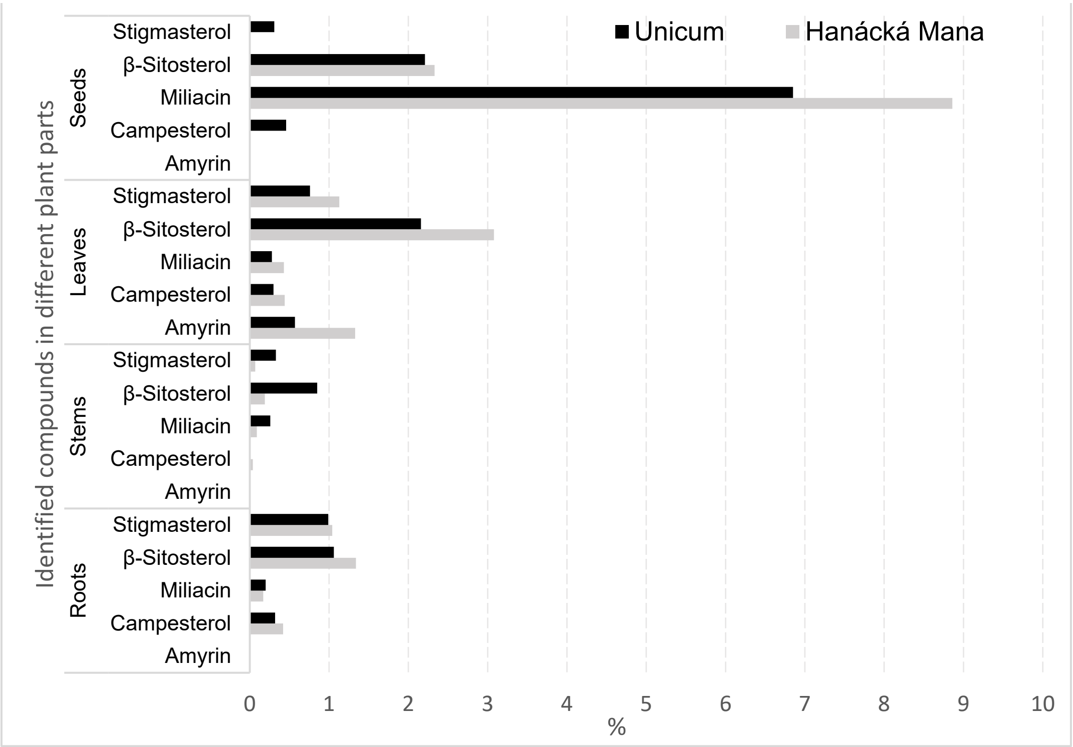

Figure S6. Comparison of the relative representation of the areas of individual peaks of identified phytosterols, amyrin and miliacin to the total area of all peaks (%) in varieties Hanácká Mana and Unicum of proso millet.

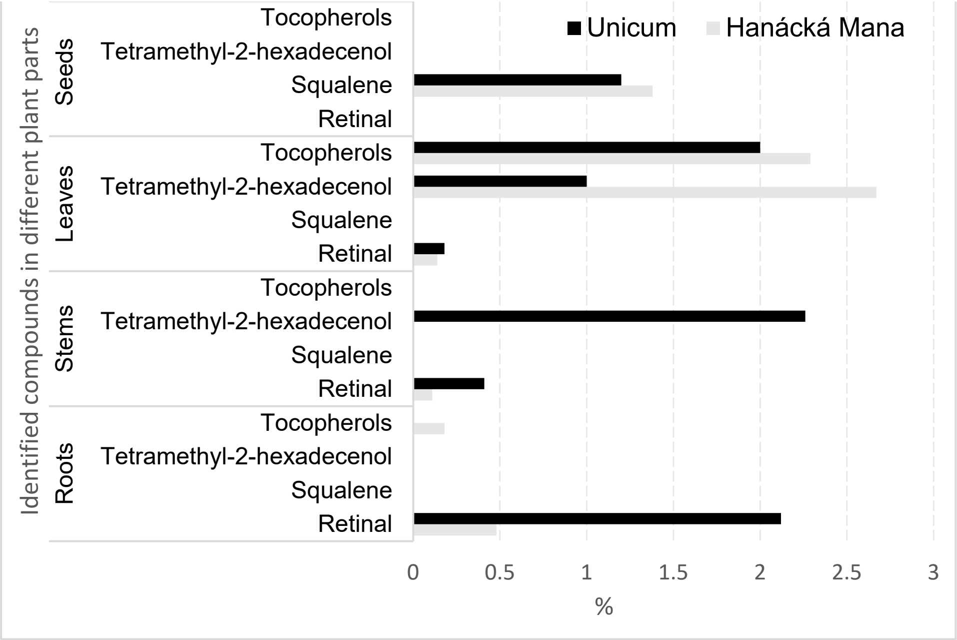

Figure S7. Comparison of the relative representation of the areas of individual peaks of identified miscellaneous compounds to the total area of all peaks (%) in varieties Hanácká Mana and Unicum of proso millet.
